# Supplementary material for: Ramosin: The First Antibacterial Peptide Identified on Bolitoglossa ramosi Colombian Salamander
Source: Pharmaceutics. 2022 Nov 23;14(12):2579. doi: 10.3390/pharmaceutics14122579 (PMC9782819; doi:10.3390/pharmaceutics14122579)
Supplement: Supplementary file 1 [file pharmaceutics-14-02579-s001.zip › pharmaceutics-2013208-supplementary.pdf]

Supplementary information

Table S1. Primers used to amplify cDNA that encoded candidate peptides of *B. ramosi.*

Candidate peptides	Forward (5′→3′)	Reverse (5′→3′)	PCR product length(bp)
AP01476	GGACGTGGAGAGCTGTCATC	CCTTCACATCTGGATACTTCTTC	441
AP02319	CAATCCGCAGCTGTTTCTATTC	TGTGCAGTCTTGCACTTTCAG	315
AP00262	CAAAGATTTCCAGTCACACAAAG	ATTCGAAAGGCTTTGTTTAGGTC	414
AP00832	GAAAGTGCTCAGAAATGCACAG	GGCAAGTTTGAGAACGCCGCCCAG	327
AP00703	TGGAATTAGAGATCATTTTGGTC	ACATCTTGGTCAATTTCCCAG	435
AP00061	GCAACATAAAGAGGGTGCTATTG	TGTAGGCCACCGTCCACTAG	333
Aureins*	GAGAATGGACTGCATCTCAACTTC	GATGAGAAGCTAGCTCTCAGCTTAC	488
AP01276	TTGGTGTCACTGTGGCTCAG	TCACACTTTCCCTGGGTCTTC	710
AP00012	TCTCGTATTCCTCGTGTTGGTG	TGGAACGTTCTTTCCGCTGTAG	432

*Aureins primers were used to amplify three candidate peptide sequences.

a.

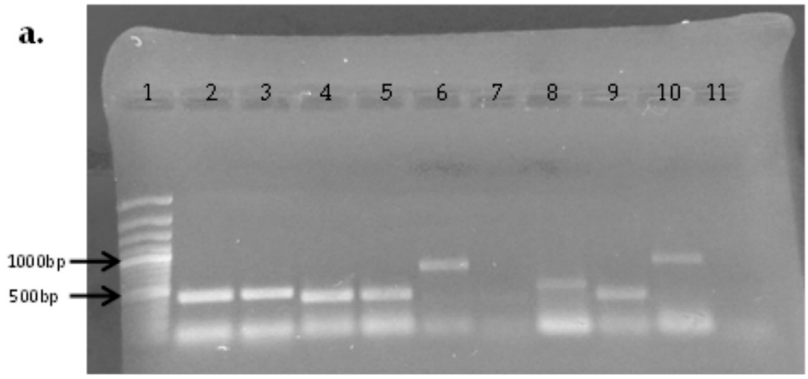

1. Ruler 1kb
2. Cholecystokinin ext 4
3. Cholecystokinin ext 7
4. Buforin ext 4
5. Buforin ext 7
6. AP01276 ext 6
7. AP02319 ext 6
8. AP01476 ext 6
9. AP00061 ext 6
10. 4174 ext 6
11. Blank

Figure S1. Amplification of the DNA sequences that encode for the candidate peptides of *Bolitoglossa ramosi*. PCR products in a 1% agarose gel stained with ethidium bromide correspond to Cholecystokinin (418 bp), Buforin (365 bp), AP01276 (710 bp), AP02319 (315 bp), AP01476 (441 bp), AP00061 (333 bp), and 4174 (755 bp). Ext and number indicate extremity and three different of *B. ramosi* specimens, respectively.

```
REVERSA_MACROGEN      ---------------------------TTTTTATTTTT----------------------
TR73493_c0_g1_i1      GATGAGAGAAGCTAGCTCTCAGCTTACTTTTTACATTTCTGTCTTTTTGTGTTAATTATG
                                                 ******  ***

REVERSA_MACROGEN      ------------------------------------------------------------
TR73493_c0_g1_i1      ACGCTGTAAGCAGCAAAATAATGTCAACAATCCACTAAACTAGTCCATCCACTTATTCCT


REVERSA_MACROGEN      ------------TGAACTTATCCATTTACTACCCTCACCACCATCCTGGCAAGCCTATTG
TR73493_c0_g1_i1      TACTTCATCCAGTGAACCTATCCATCTACTACCCTCACCACCATCCTGGCAAGCCTATCG
                                  ***** ******* ******************************** *

REVERSA_MACROGEN      TTCCACCCATTACCCTTGTCAGGCTACCTACACCTCCAACTACTTTTCTCACCAGACTAA
TR73493_c0_g1_i1      TTCCACCCATTACCCTTGTCAGGCTACCTACACCTCCAACTACTTTTCTCACCAGACTAA
                      ************************************************************

REVERSA_MACROGEN      ACCAACCCACGCACTTGCCAGCCTCGCTCTCTGTCTCTTGCTCTCGTTGTTTCTTTCCTT
TR73493_c0_g1_i1      ACCAACCCACGCACTTGCCAGCCTCGCTCTCTGTCTCTTGCTCTCGTTGCTTCTTTCCTT
                      ************************************************* **********

REVERSA_MACROGEN      CCCTCTTTTCCTTTTCCCTCGCTCTTGCTCCTGCTCTCTCTCCCTTTTTCCTTCTCTCCC
TR73493_c0_g1_i1      CCCTCTTTTCCTTTTCCCTCGCTCTTGCTCCTGCTCTCTCTCCCTTTTTCCTTCTCTCCC
                      ************************************************************

REVERSA_MACROGEN      TCGCTCCTTCGCTCCCTCCAGTGAAGAAGGGTAGATATCTTGACAGTACTGTGACTGGAC
TR73493_c0_g1_i1      TCGCTCCTTCGCTCCCTCCAGTGAAGCAGGGTAGATATCTTGACAGTACTGTGACTGGAC
                      ************************** *********************************

REVERSA_MACROGEN      TGGTAGGGCTCATAGCTGTCGTCTGGGTTTTTTTAGCCCCAAAAGAAGTTGAGATGCAGC
TR73493_c0_g1_i1      TGGTAGGGCTCATAGCTGTCGTCTGGGTTTTTTTAGCCCCAAAAGAAGTTGAGATGCAGT
                      ***********************************************************

REVERSA_MACROGEN      CCATTC---
TR73493_c0_g1_i1      CCATTCTCT
                      ******
```

Figure S2. MUSCLE alignment of the DNA sequences encoding for peptide 3412. PCR products were sequenced on Macrogen. The alignment shows the PCR product (REVERSA_MACROGEN) with the sequence in the transcriptome (TR73493_co_g1_i1). Asterisks indicate nucleotides that are conserved in both sequences. The empty spaces correspond to nucleotide changes between both sequences. The red box highlights the nucleotide sequence that codes for mature peptide 3412.

Ramosin peptide (code 3412)

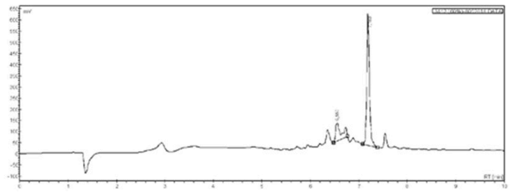

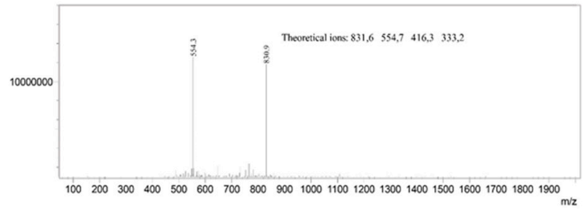

Peptide 3413

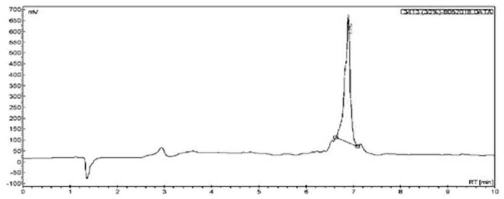

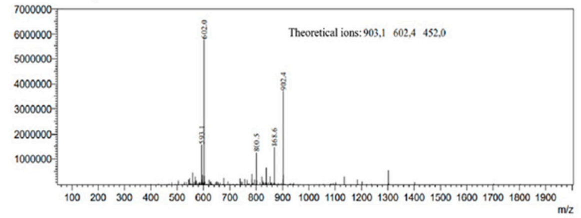

Peptide 3414

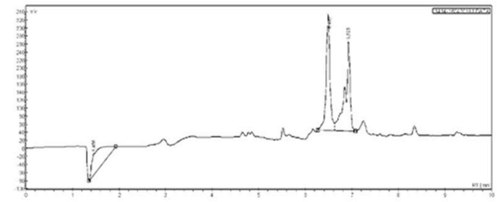

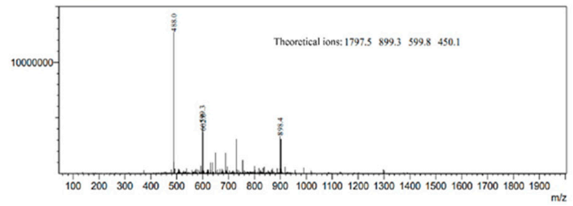

Peptide 3415

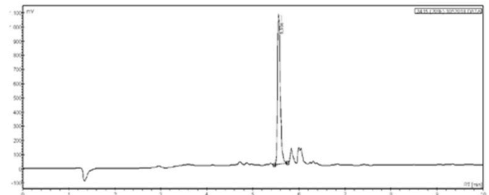

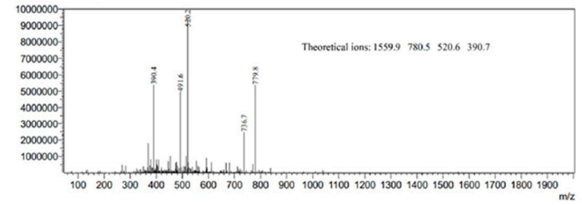

Peptide 3416

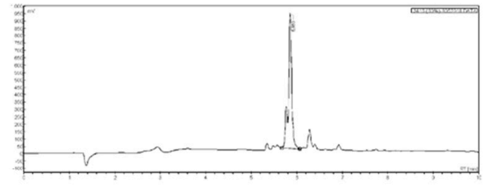

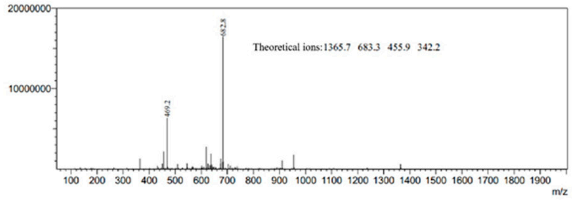

Peptide 3417

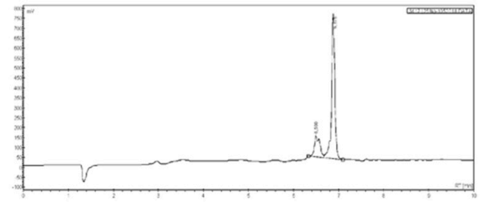

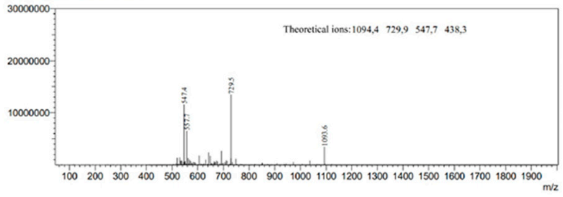

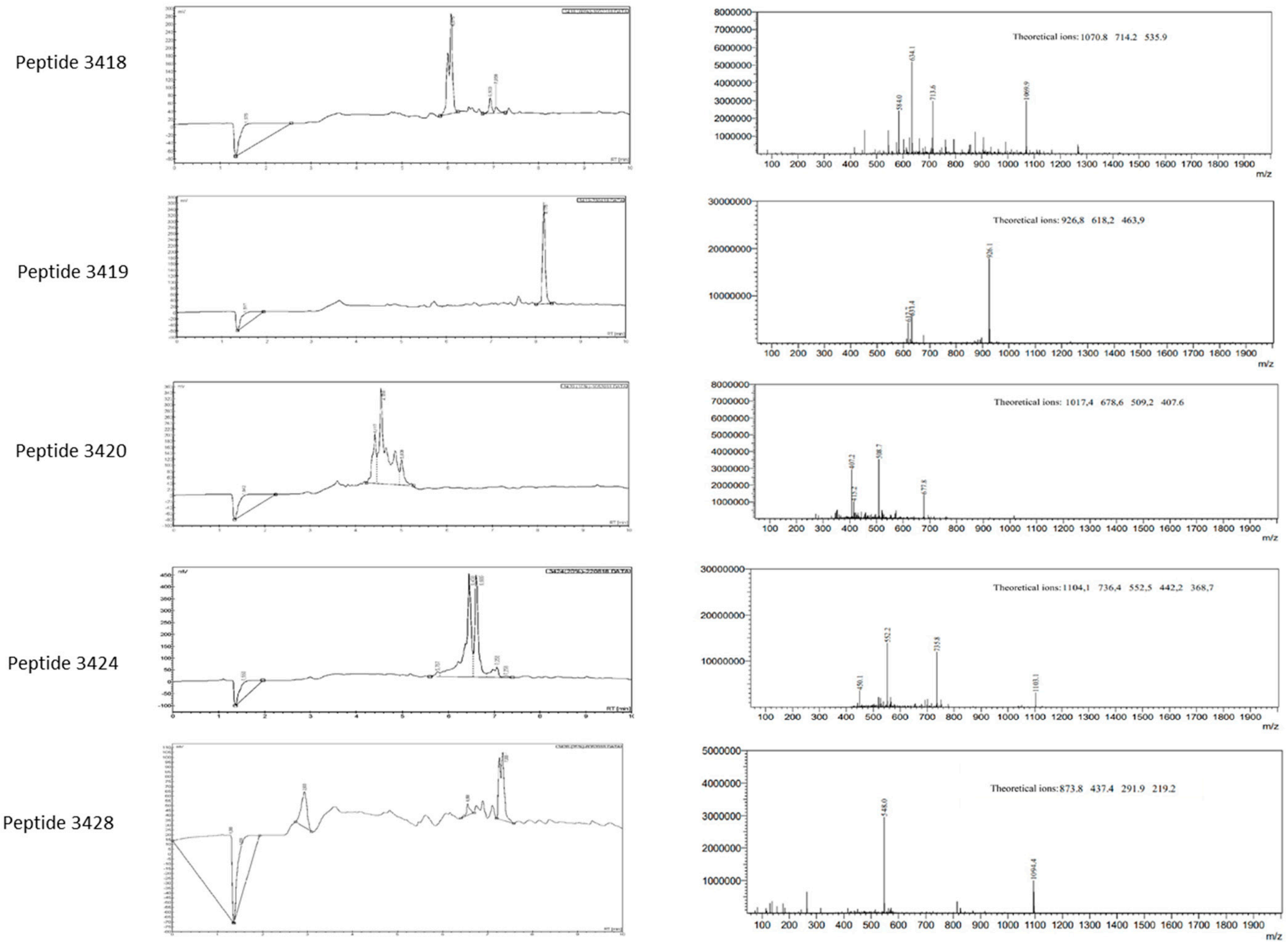

Figure S3. RP-HPLC (left) and ESI-MS (right) mass spectra of the *B. ramosi* synthetic peptides.

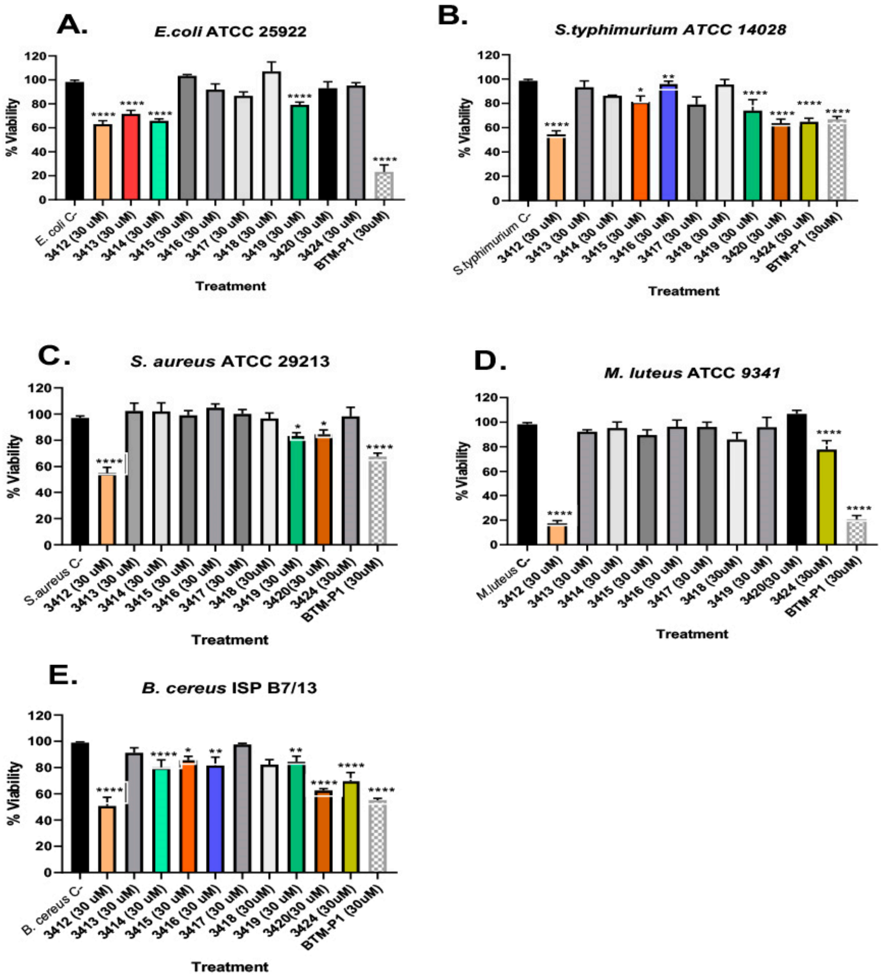

Figure S4. Viability of Gram-positive and Gram-negative bacteria were obtained in the screening test. **(a)** *Escherichia coli* **(b)** *Salmonella typhimurium* **(c)** *Staphylococcus aureus* **(d)** *Micrococcus luteus* **(e)** *Bacillus cereus.* The * show the peptides that show a significant difference in the percentage of viability of the bacteria. (* p=0.0357; ** p= 0.0099; **** p≤0.001). Three independent experiments were carried out for each bacterial strain. Data are presented as mean ± SD. The columns of the peptides that presented statistical differences, compared with the untreated bacteria, were stained. In gray boxes, peptide BTM-P1 is observed, which was used as a positive control for death.

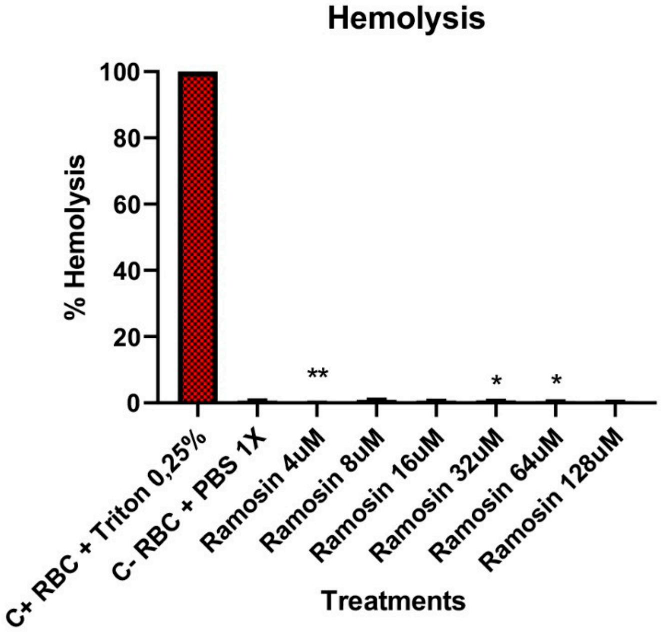

Figure S5. Hemolytic activity of the Ramosin peptide. RBC was treated for 2 hours with Ramosin at 8 μM, 16 μM, 32 μM, 64 μM and 128 μM. **C+** positive hemolysis control, consisting of RBC treated with 0.25% Triton X-100. **C –** RBC without treatment (negative control).

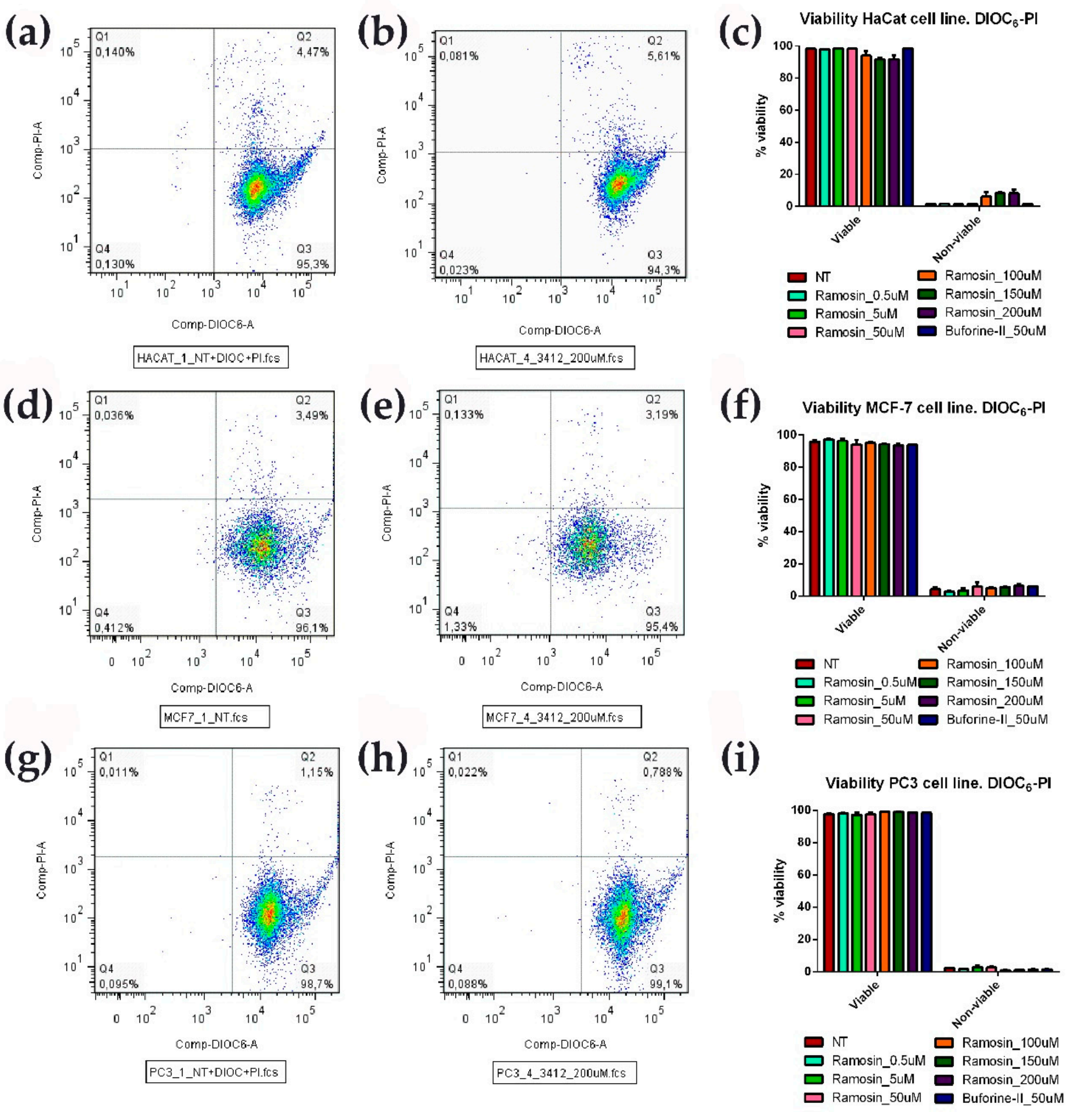

Figure S6. Determination of the percentage of cell viability and mitochondrial damage evaluated by flow cytometry using $DIOC_6$ and PI staining. The dot plots correspond to representative results of untreated cells and cells treated with the highest concentration of Ramosin peptide (200 µM) for 24h. (a),(d), (g) correspond to untreated HaCat, MCF-7, and PC-3 cells. (b), (e) and (h) correspond to the HaCat, MCF-7, and PC3 cells treated with the Ramosin peptide at 200µM and (c), (f) and (i) correspond to the histograms showing the percentage of viability of the HaCat, MCF-7 cell lines. and PC-3 treated for 24 hours with the Ramosin peptide at 0.5µm, 5µm and 50µM and Buforin II peptide at 50µM.

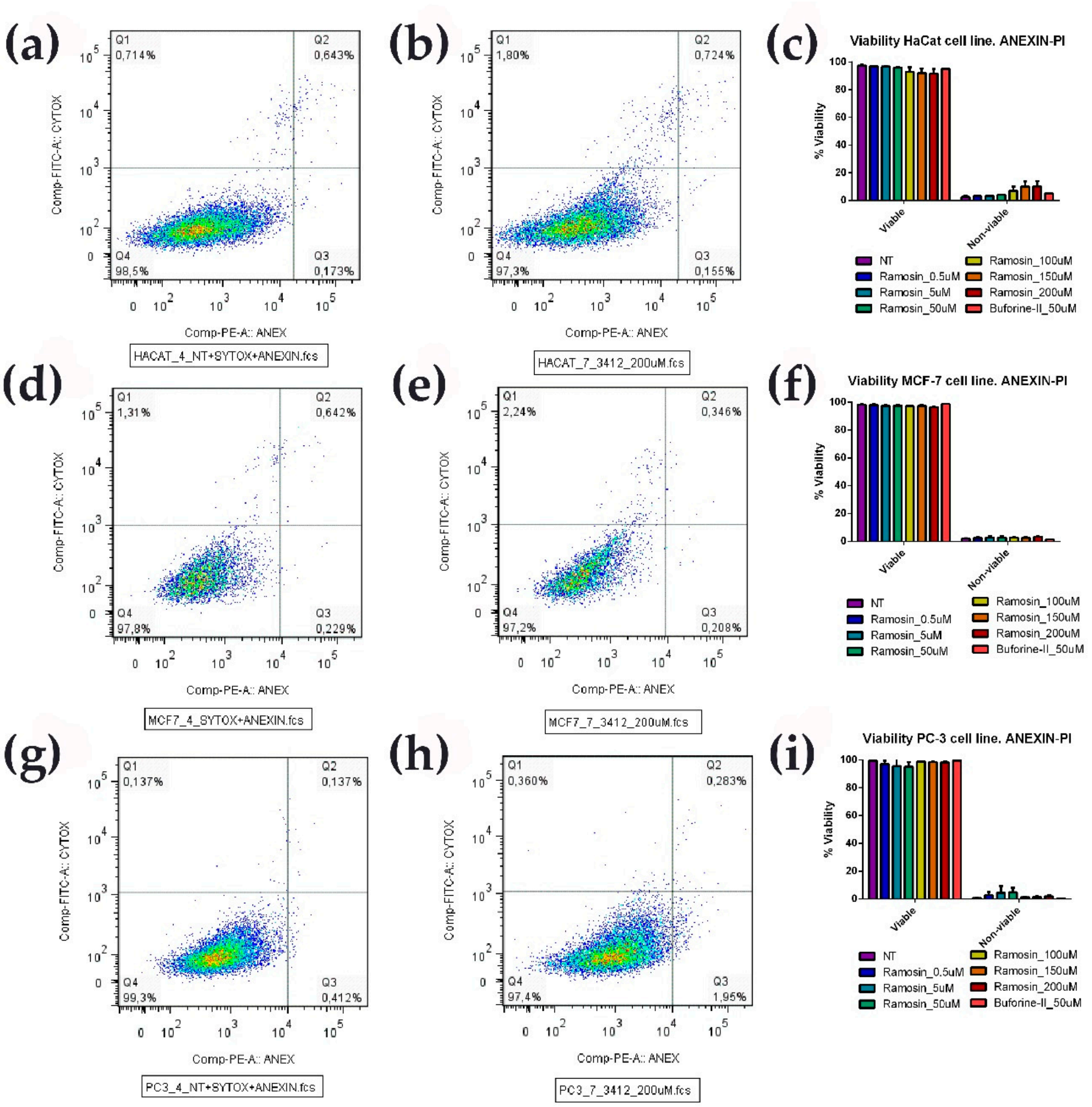

Figure S7. Determination of the percentage of cell viability evaluated by flow cytometry using a double staining with Annexin-V and SYTOX. The dot plots correspond to representative results of untreated cells and cells treated with the highest concentration of Ramosin peptide (200μM) for 24h. (a), (d), (g) correspond to untreated HaCat, MCF-7, and PC-3 cells. (b), (e), and (h) correspond to the HaCat, MCF-7, and PC3 cells treated with the Ramosin peptide at 200μM and (c), (f), and (i) correspond to the histograms showing the percentage of viability of the HaCat, MCF-7 cell lines. and PC-3 treated for 24 hours with the Ramosin peptide 3412 at 0.5 μM, 5 μM and 50 μM and Buforin II peptide at 50 μM.

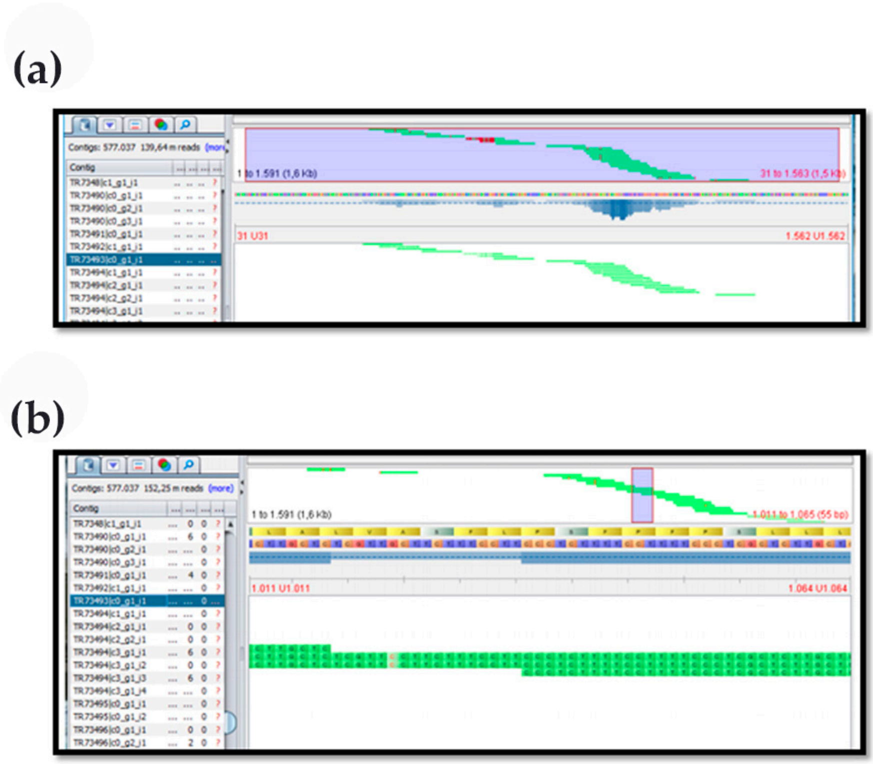

Figure S8. Distribution of the reads in the transcript TR73493_c0_g1_i1 where the Ramosin peptide was found encoded. The green lines represent each of the reads. (a) distribution of the reads (b) close-up where it can be seen that the reads overlap, guaranteeing that the assembled transcript is supported and that it was not a bioinformatic artifact.
